# Effects of the cefazolin shortage on the sales, cost, and appropriate use of other antimicrobials

**DOI:** 10.1186/s12913-021-07139-z

**Published:** 2021-10-19

**Authors:** Ryuji Koizumi, Yoshiki Kusama, Yusuke Asai, Gu Yoshiaki, Yuichi Muraki, Norio Ohmagari

**Affiliations:** 1 AMR Clinical Reference Center, Disease Control and Prevention Center, National Center for Global Health and Medicine, Tokyo, Japan; 2 Collaborative Chairs Emerging and Reemerging Infectious Diseases, National Center for Global Health and Medicine, Graduate School of Medicine, Tohoku University, Sendai, Miyagi Japan; 3 Department of Clinical Pharmacoepidemiology, Kyoto Pharmaceutical University, Kyoto, Japan

**Keywords:** Drug shortage, Appropriate antimicrobial use, Essential medicine, Cefazoline, AWaRe classification

## Abstract

**Background:**

Shortages of antimicrobials lead to treatment failures, increase medical costs, and accelerate the development of antimicrobial resistance. We evaluated the effects of the serious cefazolin shortage in 2019 in Japan on the sales, costs, and appropriate use of other antimicrobials.

**Methods:**

We evaluated monthly defined daily doses/1000 inhabitants/day (DID) values of antimicrobial sales from January 2016 to December 2019 using wholesaler’s sales databases. Using 2016–2018 sales data, we generated a prediction model of DID in 2019 under the assumption that the cefazolin shortage did not occur. We then compared the predicted DID and actual DID. Cefazolin, government-recommended alternatives, and government-not-recommended broad-spectrum alternatives were assessed. Antimicrobial groups according to the AWaRe classification were also assessed to evaluate the effect on appropriate antimicrobial use. In addition, we evaluated changes in costs between 9 months before and after the cefazolin shortage.

**Results:**

DID values of total antimicrobials increased sharply 1 month before the decrease in cefazolin. Actual DIDs were higher than predicted DIDs for ceftriaxone, flomoxef, clindamycin, cefotiam, piperacillin/tazobactam, and meropenem. Actual DID values were higher than the predicted DID values in the Watch group. The costs of antimicrobials between pre- and post- cefazolin shortage were unchanged.

**Conclusion:**

The cefazolin shortage brought confusion to the antimicrobial market and led to a setback in the appropriate use of antimicrobials. Early recognition and structures for prompt reactions to antimicrobial shortages are needed. Moreover, development of a system to secure the supply of essential antimicrobials is required.

**Supplementary Information:**

The online version contains supplementary material available at 10.1186/s12913-021-07139-z.

## Background

The sustainable supply of medical drugs is important for providing quality-assured medicine; therefore, countermeasures for preventing shortages are required [1–3]. According to a US Food and Drug Administration report, the United States has faced many drug shortages in various regions, and the shortages disturbed the use of quality-assured medicines [4]. A sustainable drug supply is also warranted for antimicrobials. Antimicrobial shortages can make first-line treatments unavailable, resulting in the use of less effective, more toxic, or more expensive alternatives [5]. In terms of treatment, narrow-spectrum antimicrobials can be compensated for by using broad-spectrum antimicrobials during shortages. However, such countermeasures increase the risk of the emergence of antimicrobial resistant organism [6]. The United States has repeatedly faced problems due to antimicrobial shortages, and their frequency has recently increased [7]. Piperacillin/tazobactam shortages increase the risk of nosocomial *Clostridioides difficile* infections [8], and it is estimated that one antimicrobial shortage results in excess costs of 2.4–3.5 million USD [9].

**Fig. 1:**
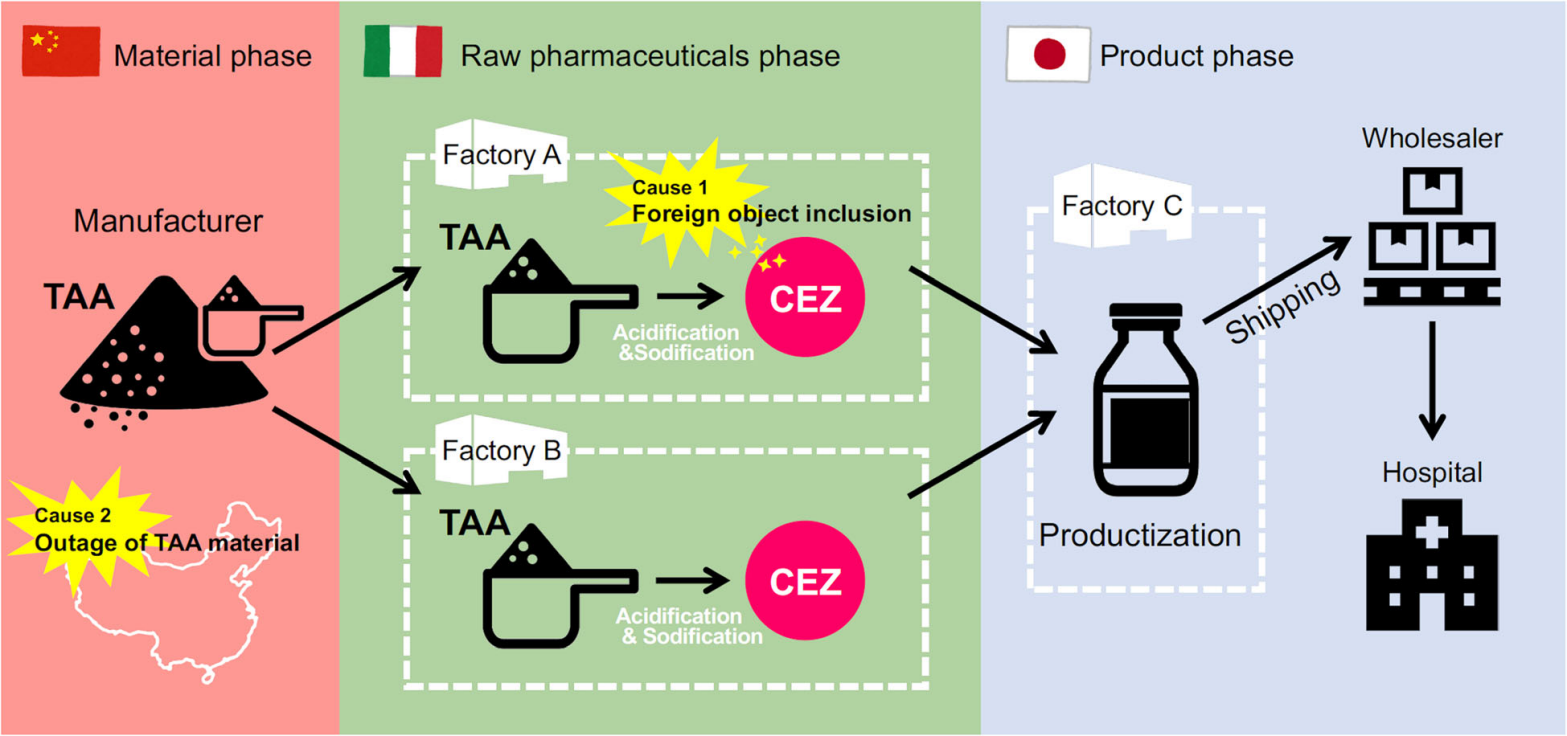
Process in the production of cefazolin in the company of supply disruption. Manufacturer provides TAT to Factory A and B. Factory A and B synthesize CEZ, then Factory C productizes it. Foreign body inclusion was found in Factory A, and the factory stopped working. Thereafter, the outage of TAA material occurred in Manufacturer. These events caused a short of materials in Factory C, and resulted in a cease of cefazolin production. Abbreviation: TAA, Tetrazole-Acetic Acid. CEZ, Cefazolin

In January 2019, the largest pharmaceutical factory producing cefazolin in Japan experienced manufacturing difficulties. The background of the shortage was shown in Fig. 1 [10]. Cefazolin is the representative first-generation cephalosporin classified as an “Access” antimicrobial (i.e., countries should maintain availability at any time and in any situation) in the WHO Model Lists of Essential Medicines [11, 12]. It is often used for the treatment of methicillin-sensitive *Staphylococcus aureus* infections and many kinds of surgical prophylaxis [13–15].

In Japan, cefazolin is the second most widely used parenteral antimicrobial followed by ceftriaxone, and the most used antimicrobial among inpatients [16]. The Ministry of Health, Labour and Welfare of Japan published a list of alternative antimicrobials to cefazolin [17]; however, this caused secondary shortages among the alternative drugs [18]. Collapse of antimicrobial stewardship due to the cefazolin shortage has already been reported by a single center, and demonstrated the rapid increase in the use of third-generation cephalosporins after the cefazolin supply became limited [19].

The aim of this study was to clarify the effect of cefazolin shortages on the supply of other antimicrobials by observing trends in antimicrobial sales at the national level, and to evaluate the effects on appropriate antimicrobial use and drug costs brought about by the shortage.

## Methods

### Design

Retrospective observational study using sales data of antimicrobials collected through wholesalers.

### Settings

Antimicrobial sales from 2013 to 2019 in all Japan.

### Drug pricing system in Japan

In Japan, medical reimbursement costs, which include drug costs, are standardized by the Ministry of Health, Labour and Welfare in Japan en bloc. Therefore, prices of drugs paid by insurers to medical facilities are the same under all circumstances. These reimbursement prices of drugs are reviewed based on market situations in April every 2 years. To control health care expenditures, drug reimbursement prices tend to be gradually reduced. Although drug prices were not altered in 2019, the reimbursement prices were altered outside the usual schedule in October because of a tax increase that month.

### Evaluating drug

The DID values of cefazolin, government-recommended alternatives (recommended by the Ministry of Health, Labour and Welfare of Japan; see Table 1), and non-government-recommended broad-spectrum alternatives were evaluated. Among the recommended alternatives, we analyzed commonly used drugs (DID higher than 0.01) during the study period (namely, ceftriaxone, ampicillin/sulbactam, cefmetazole, vancomycin, levofloxacin, flomoxef, clindamycin, and cefotiam). In government-not-recommended broad-spectrum alternatives, we evaluated piperacillin/tazobactam and meropenem because they are frequently used broad-spectrum parenteral antimicrobials in Japan.

**Table 1:** Government-recommended alternatives

Treatment		
Disease	Parenteral antimicrobial	Oral antimicrobial
Sepsis caused by methicillin-sensitive *Staphylococcus aureus*	Ampicillin and sulbactam	
	Cefotaxime	
	Ceftriaxone	
	Vancomycin	
	Daptomycin	
Soft tissue infection (e.g., cellulitis,erysipelas)	Ampicillin and sulbactam	Amoxicillin and clavulanate
	Cefotaxime	Cephalexin
	Ceftriaxone	Clindamycin
	Clindamycin	
Acute osteomyelitis / septic arthritis	Ampicillin and sulbactam	
	Cefotaxime	
	Ceftriaxone	
	Vancomycin	
	Daptomycin	
Urinary infection (acute pyelonephritis)	Cefotiam	Ciprofloxacin
	Cefmetazole	Levofloxacin
	Flomoxef	Sulfamethoxazole and trimethoprim
	Cefotaxime	
	Ceftriaxone	
	Aminoglycosides	
Prophylaxis		
Surgical cite / bacteria	Parenteral antimicrobial	Oral antimicrobial
Neuro surgery/*Staphylococcus aureus*, Streptococcus	Cefotiam	
	Cefotaxime	
	Ceftriaxone	
	Clindamycin	
	Vancomycin	
Otorhinolaryngology/*Staphylococcus aureus*, Oral anaerobic bacteria, Streptococcus	Ampicillin and sulbactam	
	Cefotiam	
	Clindamycin	
Cardiovascular surgical procedure (heart, vessel)/*Staphylococcus aureus*, Streptococcus	Cefotiam	
	Cefotaxime	
	Ceftriaxone	
	Clindamycin	
	Vancomycin	
thoracic surgery (lung, trachea)/Oral anaerobic bacteria, Streptococcus	Ampicillin and sulbactam	
	Cefotiam	
	Clindamycin	
Breast surgery/*Staphylococcus aureus*, Streptococcus	Cefotiam	
	Clindamycin	
Upper Gastrointestinal Tract Surgery (esophagus, stomach, jejunum) /*Escherichia coli*, *Klebsiella pneumoniae*	Ampicillin and sulbactam	
	Cefotiam	
Gastrointestinal surgery (liver, gallbladder, bile duct, pancreas)/Enterobacteriaceae	Ampicillin and sulbactam	
	Cefotiam	
	Cefmetazole	
	Flomoxef	
Gynecology/Enterobacteriaceae, Group of *Bacteroides fragilis*	Ampicillin and sulbactam	
	Cefotiam	
	Cefmetazole	
	Flomoxef	
Urology/Enterobacteriaceae	Ampicillin and sulbactam	Ciprofloxacin
	Cefotiam	Levofloxacin
	Aminoglycoside	
	Ciprofloxacin	
	Levofloxacin	
Orthopedic surgery (Spinal Surgery, Artificial Bone Replacement, etc)/*Staphylococcus aureus*, Streptococcus	Cefotiam	
	Cefmetazole	
	Flomoxef	
	Clindamycin	
	Vancomycin	

### Assessing temporal trends in sales

We evaluated monthly sales volumes of parenteral antimicrobials and analyzed the distribution of antimicrobials in Japan. Sales volume was measured using defined daily doses according to the WHO Collaborating Center for Drug Statistics Methodology, and was represented as defined daily doses (DDDs)/1000 inhabitants/days (DID) [20]. The equation used is shown below.

$$ \mathrm{DDDs}/1000\ \mathrm{inhabitants}/\mathrm{day}\ \left(\mathrm{DID}\right)=\frac{\mathrm{Sales}\ \mathrm{of}\ \mathrm{each}\ \mathrm{month}\ \left(\mathrm{g}\right)}{\mathrm{DDD}\ \left(\mathrm{g}\right)\times \mathrm{population}\ \mathrm{of}\ \mathrm{each}\ \mathrm{year}\ \left(/1000\ \mathrm{inhabitants}\right)\times \mathrm{days}} $$

We defined antimicrobials as code J01 according to Anatomical Therapeutic Chemicals developed by WHO Collaborating Centre for Drug Statistics Methodology [21]. We evaluated the temporal trend of monthly DID values of total antimicrobials and cefazolin in 2019 compared with the previous 3 years.

### Comparing sales between the real and predicted data

We predicted DID values in 2019 by using DID values from 2013 to 2018 under the assumption that a cefazolin shortage did not occur, and compared real DID values. Prediction models were formulated using the seasonal autoregressive integrated moving average (SARIMA) model.

### Assessing appropriateness

To assess the appropriateness of antimicrobial use, we created models of aggregate total antimicrobial sales, and of “Access”, “Watch”, and “Reserve” antimicrobials according to AWaRe classification. Predicted monthly sales were compared with actual monthly sales in 2019 by using box plots. We defined “appropriate” as an increase in drugs categorized under “Access” and “inappropriate” as an increase in drugs categorized under “Watch”.

### Assessing drug costs

We evaluated the changes in drug costs between before and after the cefazolin shortage. Durations of analysis were set as the 9 months before (April to December 2018) and after (January to September 2019) the disruption in cefazolin manufacturing, because drug prices did not change in these periods. Additionally, to adjust for seasonal variations, we generated linear regressions of costs on DDD values in total antimicrobials, and compared the difference of the co-efficient in regression equations.

### Data source

IQVIA Japan is a company that provides a database of pharmaceutical information obtained from wholesalers to researchers and companies. Although patient information is not included in this database, it stores data about all medical drugs sold by wholesalers to medical facilities. Almost all medical facilities in Japan purchase drugs through wholesalers, so this database is considered to be representative of national data. We purchased the database and retrospectively analyzed the data [22].

### Statistics

We performed all statistical analysis using R ver.4.0.0 (R Foundation for Statistical Computing, Vienna, Austria). The Forecast package was employed to conduct predictions based on the SARIMA model.

### Ethics

The need for ethics review was waived because all data were anonymized before we obtained it.

## Results

### Temporal trends in sales

**Fig. 2:**
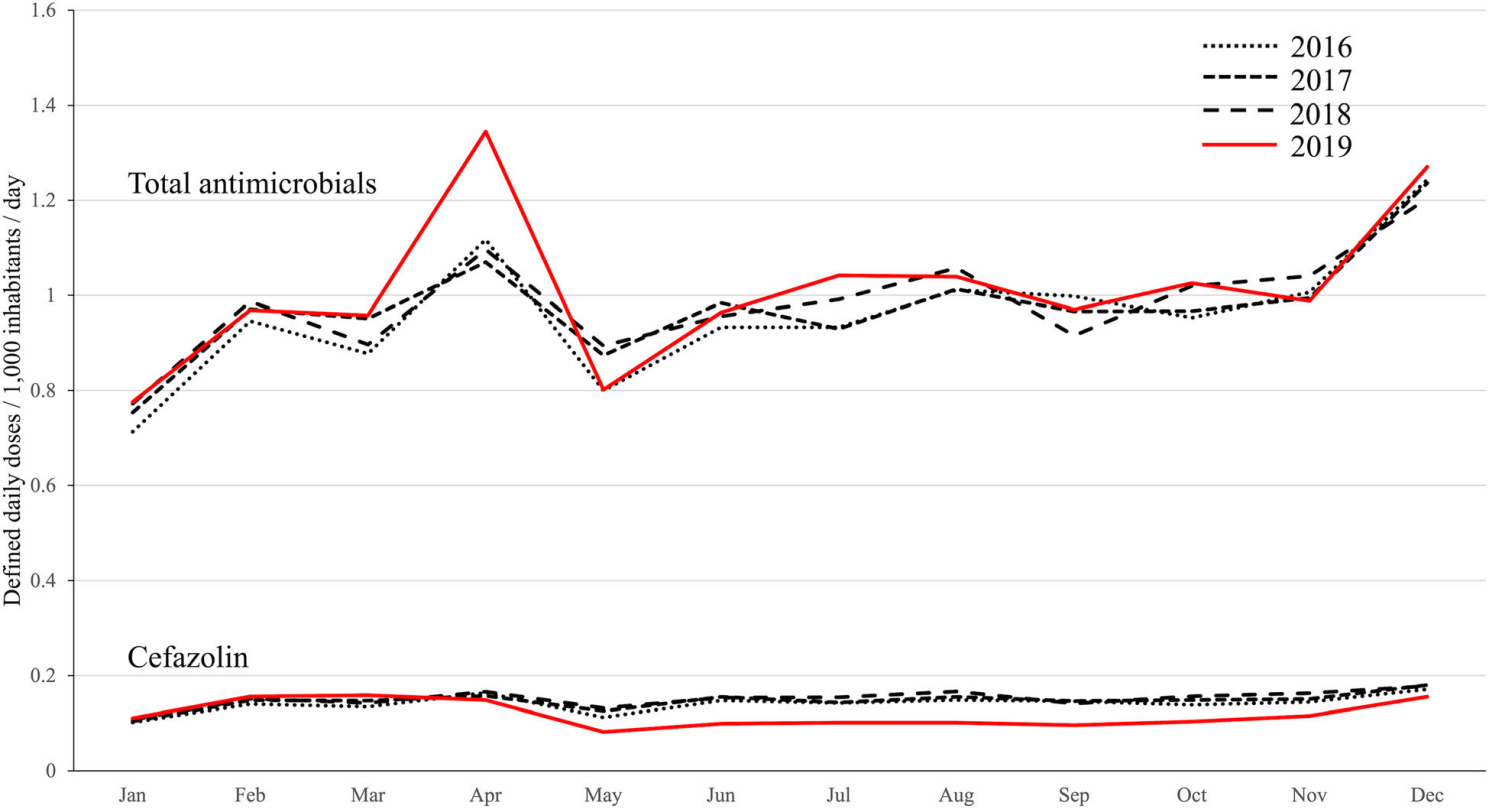
Monthly DID values of parenteral antimicrobials from 2016 to 2019. The solid line represents DID values in 2019. The DID value of cefazolin decreased in May, and the DID value increase for total antimicrobials was observed one month before the decrease in cefazolin. Abbreviation: DID, defined daily doses/1000 inhabitants/day

The pharmaceutical company with the largest share of cefazolin in Japan declared a halt to manufacturing on February 28 and production stopped in early March. The DID value of cefazolin decreased from April to May due to the exhaustion of distribution stock. Cefazolin sales declined in April and reached their lowest point in May. This low level of sales continued until November 2019, after which sales gradually began to recover. Meanwhile, the DID value of total parenteral antimicrobial sales in April 2019 was 1.35, which was the highest DID value in 4 years (1.07–1.10). From May 2019, the DID value returned to a value comparable to that in the previous 3 years (Fig. 2).

### Comparison between the real and predicted data

**Fig. 3:**
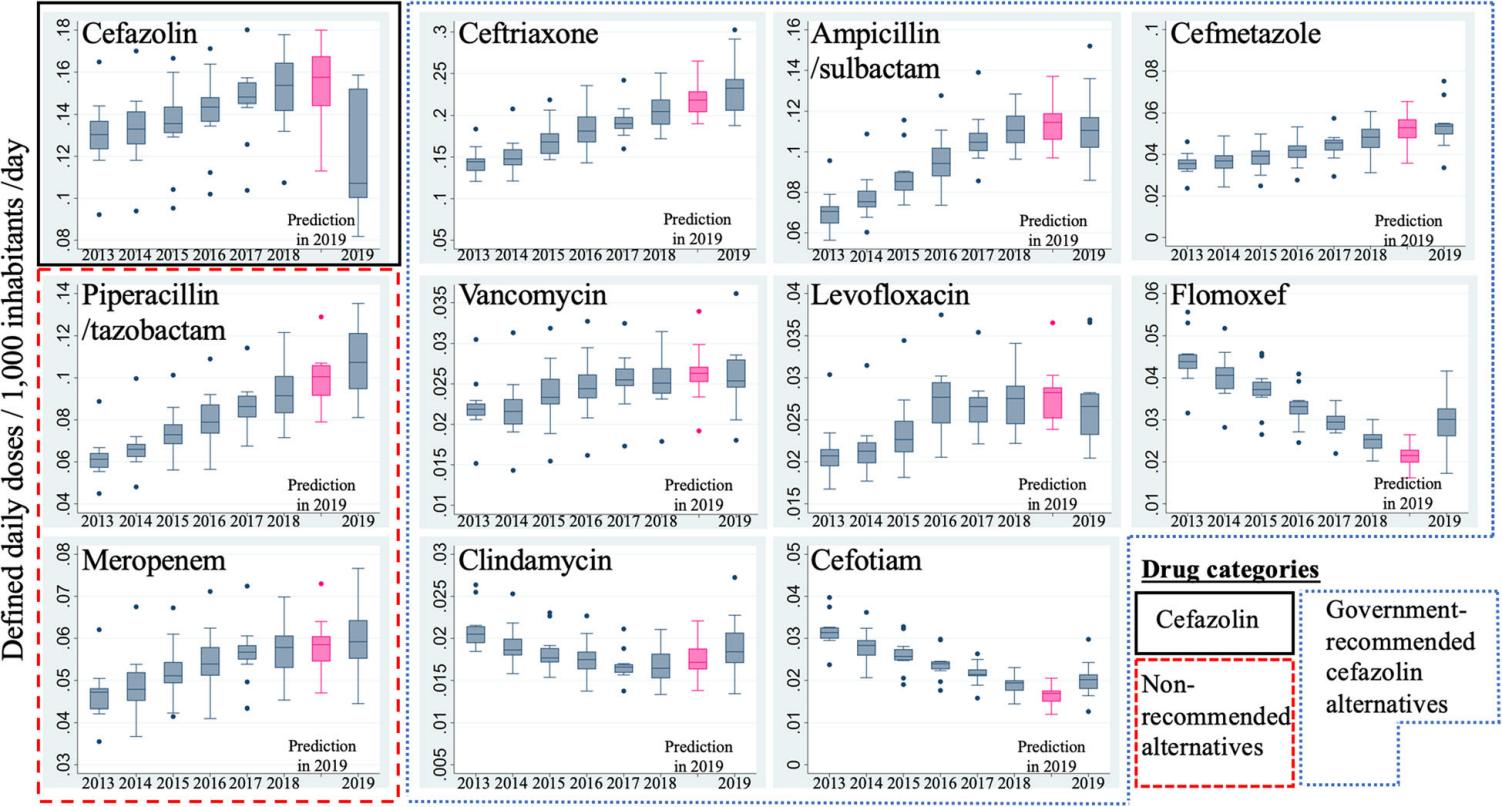
Box plots of actual and predicted DID values of antimicrobials. Annual trends in box plots of monthly DID values are shown. Light gray box plots represent predictions of DID values in 2019 generated by 2013–2018 data under the assumption of non-occurrence of the cefazolin shortage. Solid line squares represent cefazolin, fine dashed lines represent government-recommended cefazolin alternatives, and red dashed lines represent government-not-recommended broad-spectrum cefazolin alternatives. Abbreviation: DID, defined daily doses/1000 inhabitants/day

Monthly DID values of selected antimicrobials from January 2013 to December 2019 and predicted DID values predicted by the SARIMA model under the assumption of the absence of a cefazolin shortage are shown in Supplementary Figs. 1 and 2. Figure 3 shows the annual trends in box plots of monthly DID values, including the 2019 predictions. Compared with the prediction box, the actual box was lower for cefazolin in 2019. Among government-recommended alternatives, ceftriaxone, flomoxef, clindamycin, and cefotiam showed higher box plots of actual DID values than the predicted values. In contrast, ampicillin/sulbactam, cefmetazole, vancomycin, and levofloxacin showed no obvious increases in the box plots of actual DID values compared with the predictions. Among government-not-recommended broad-spectrum alternatives (e.g., piperacillin/tazobactam and meropenem), the box plots of actual DID values were higher than those of the predictions.

### Appropriateness

**Fig. 4:**
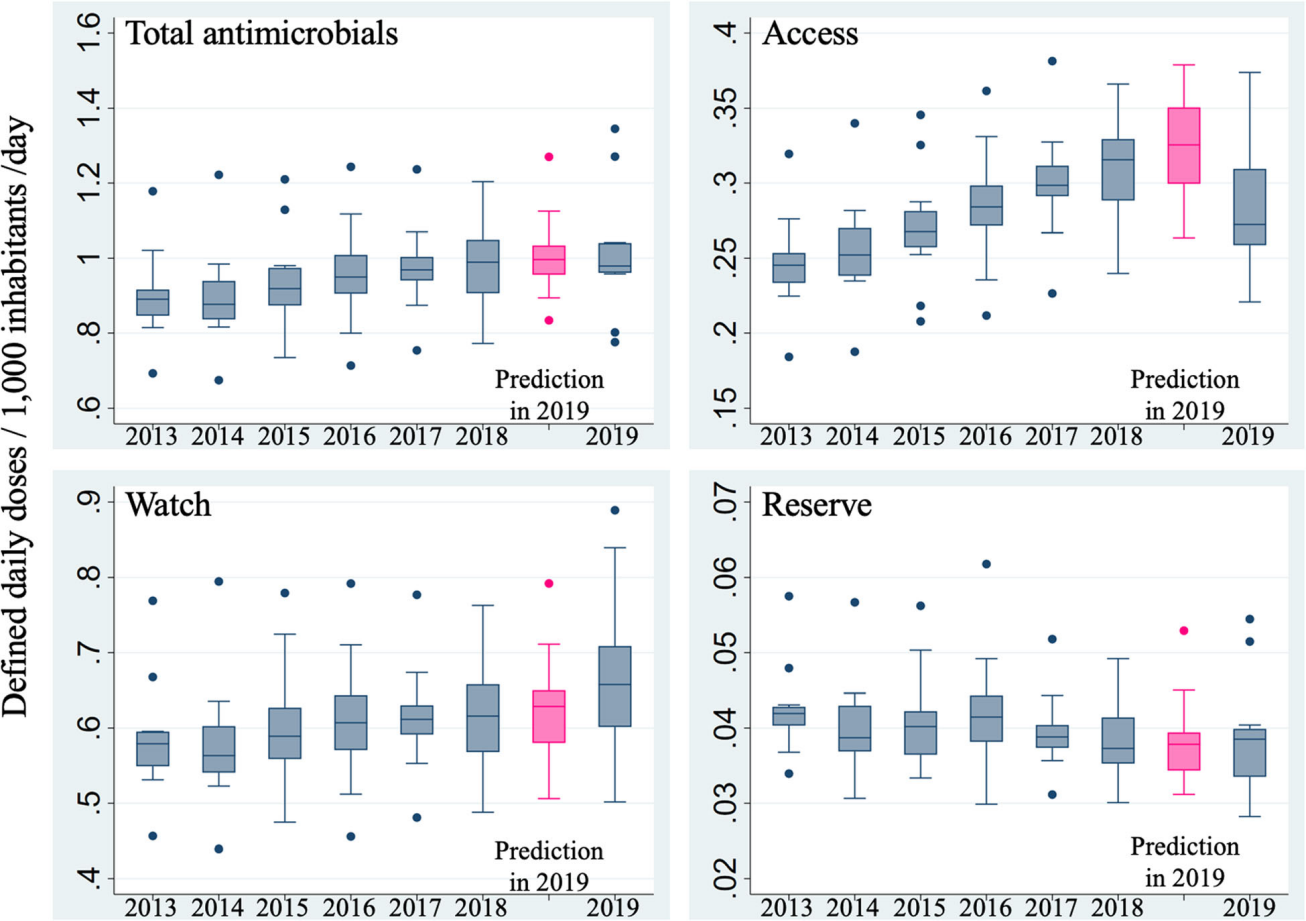
Box plots of actual and predicted DID values of antimicrobials according to AWaRe classifications. Annual trends in box plots of monthly DID values are shown. Light gray box plots represent predictions of DID values in 2019 generated by 2013–2018 data under the assumption of non-occurrence of the cefazolin shortage. Abbreviation: DID, defined daily doses/1000 inhabitants/day

Similarly, total sales of parenteral antimicrobials grouped by the AWaRe classification are shown in Fig. 4. Actual DID values of total parenteral antimicrobials did not show large differences compared with the predictions. Meanwhile, actual sales in the Access group were lower than predicted, and those in the Watch group were higher than predicted.

### Drug cost

**Fig. 5:**
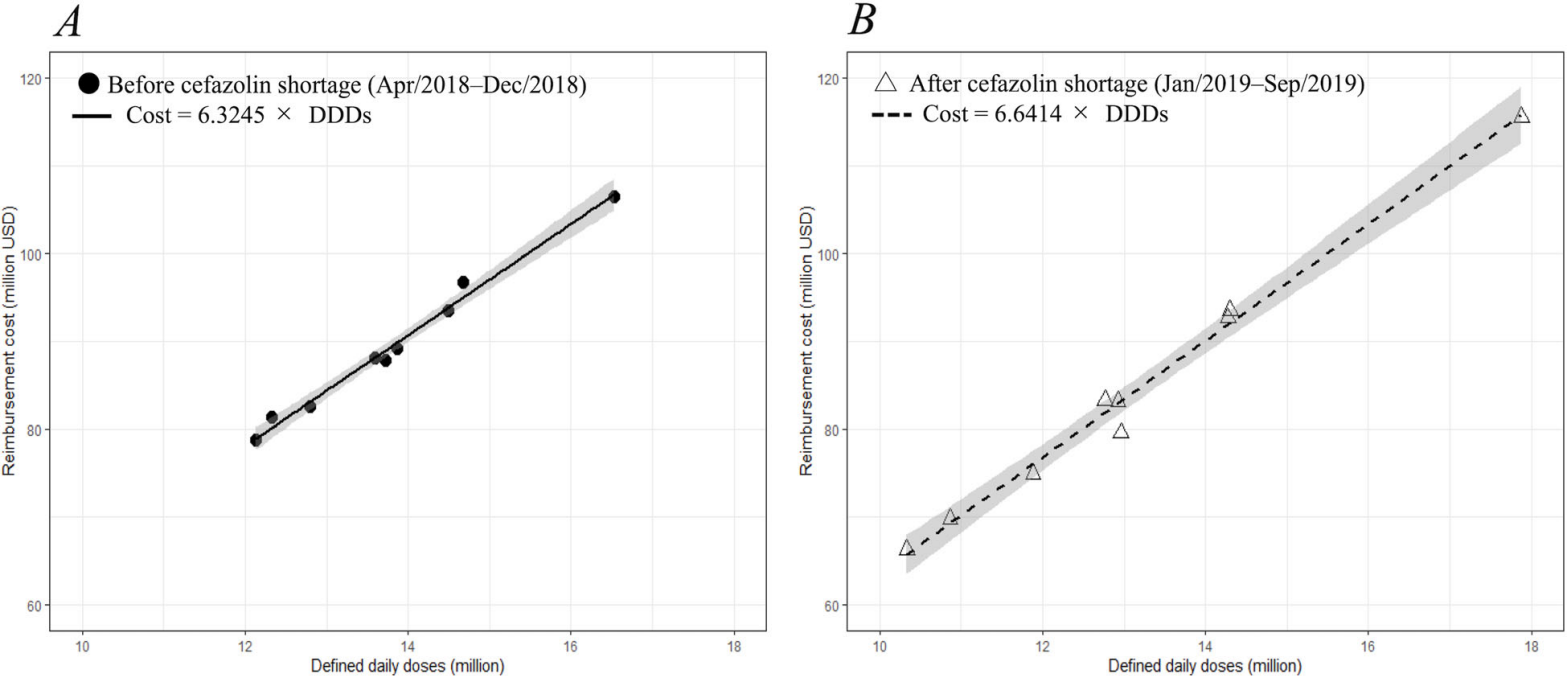
Linear regressions of reimbursement costs on defined daily doses in total parenteral antimicrobials before and after the cefazolin shortage. *A*, Black dots represent April to December 2018 (before the cefazolin shortage). *B*, White triangles represent January to September 2019 (after the cefazolin shortage). Gray zones represent 95% confidence intervals. Coefficients of formulas between A and B were similar, thus, it is considered that the reimbursement cost per defined daily dose did not change between before and after the cefazolin shortage

No differences in the ratio of total costs to total DID values were observed from 9 months before and after the cefazolin shortage (Fig. 5).

## Discussion

Our study highlighted that the cefazolin shortage led to a steep increase in total antimicrobial sales due to the rush to secure alternative antimicrobials to cephazolin. The shortage also led to an increase in the use of broad-spectrum antimicrobial agents, which in turn led to inappropriate use of antimicrobials. The cefazolin shortage highlighted various aspects of problems with the drug supply, including a deficiency in the management of distribution, inadequate supply of alternative antimicrobials, and regulations to prevent the overuse of non-recommended alternatives. Meanwhile, the basic drug production system and low drug prices have caused many of these issues.

First, the shortage drew attention to the inadequate management of drug distribution.

Temporal changes in DID values clearly showed that the shortage of essential antimicrobial brought confusion to the antimicrobial market. The cefazolin DID values decreased from April to May. Most Japanese hospitals buy drugs through wholesalers; therefore, wholesalers’ stockpiles might have resulted in this lag. Meanwhile, the total antimicrobial DID values increased from March to April, which was 1 month before the decline in the manufacture of cefazolin. This suggested that the increase in total antimicrobial sales was not brought by the cefazolin shortage itself, but by information provided on the coming shortage. The information resulted in hospitals stocking up on antimicrobials. From this perspective, action by the Ministry of Health, Labour and Welfare of Japan was later than when stocking up took place. This highlighted the importance of early recognition and the need for structures for an appropriate governmental response when a shortage occurs. We consider that the government should have moved more quickly and decisively to take control of the distribution. Accordingly, to move more quickly, the government, companies, and hospitals need to maintain a close connection, and exchange information with each other. Also, frameworks for avoiding reckless purchasing by hospitals and to secure the proper distribution of medical resources are needed.

Second, the inadequate supply of alternative antimicrobials and the lack regulations to prevent the overuse of non-recommended alternative are also problems that were revealed by the shortage. The cefazolin shortage also affected DID values of other antimicrobials. The actual DID values of the government-recommended alternatives ceftriaxone, flomoxef, clindamycin, and cefotiam were higher than their predicted DID values. It was suggested that drug makers increased the production of these drugs to alleviate damage caused by the cefazolin shortage; however, the production increases were insufficient to compensate for the cefazolin shortage, which led to secondary shortages among these drugs [19]. According to a questionnaire-based study, the cefazolin shortage caused considerable damage in the maintenance of quality-assured medicines in various hospitals [23]. Furthermore, antimicrobial surveillance in Japan in 2017 showed that 5 drugs—ceftriaxone, cefazolin, ampicillin/sulbactam, piperacillin/tazobactam, and meropenem—accounted for 59.2% of total parenteral antimicrobial sales [16]. Increases in DID values among government-not-recommended alternatives, such as piperacillin/tazobactam and meropenem, may be explained by the extra production capacity that was brought about by their superiority in the market. However, unlike meropenem and piperacillin/tazobactam, ampicillin/sulbactam was not increased. The reason for this might be due to a lack of extra production capacity (ampicillin/sulbactam have been subject to supply issues several times in the past). Vancomycin was not also increased, but the reason for this is considered to be that physicians in Japan do not routinely use vancomycin for treatment or prevention of infectious diseases. Comparison by AWaRe group between actual and predicted DID values showed a decrease in “Access” antimicrobials and an increase in “Watch” antimicrobials. This means more broad-spectrum antimicrobials were used than narrow-spectrum ones because of the cefazolin shortage, meaning that the shortage appeared to harm appropriate antimicrobial use.

Since materials of domestically used antimicrobials depend on imports from China or India, it is difficult to improve the vulnerability of the supply chain by domestic measures alone [18, 24]. Currently, problems in antimicrobial factories in China or India have caused critical damage to the supply chain in developed countries; these are similar issues irrespective of the country. Likewise, this cefazolin shortage was caused by a Chinese government requirement pertaining to environmental protection and a temporary order to stop factories. From a short-range view, it is important to secure multiple antimicrobial resources for risk management to maintain a sustainable antimicrobial supply. From a long-range view, developed countries may need to develop domestic production of antimicrobial resources, but this requires support at the national or international level.

Finally, systems in drug pricing and production are the problems. Despite the cefazolin shortage, the cost of antimicrobials per sales barely changed. This is thought to be because most antimicrobials used in Japan are generic drugs, and thus the cost differences between cefazolin and alternative drugs were small. Moreover, another important reason is that a cefazolin price increase did not occur because the reimbursement price of drugs is fixed by the government. Price setting is an important tool for securing the economy of medicine. However, reimbursement prices of drugs are gradually reduced year by year, which reduces the profit margins of pharmaceutical companies, especially for older drugs. This trend was accelerated by a recent increase in production cost s[4, 25, 26]; cefazolin is also in the same situation. Low profits disrupt the production of affordable drugs and may lead to quality deterioration due to loss of investment. To maintain the stable production of essential medicines, the government should determine the appropriate drug price to sufficiently maintain companies’ investments. In the development of new antimicrobials, delinking profits and sales, for example, through government purchasing of drugs or/and the adoption of a subscription model, is desirable from a long-term perspective.

Our study has several limitations. First, because sales data can only clarify product circulation, the actual usage of antimicrobials was not assessed in this study. Different approaches such as the use of claims data or/and hospital data are needed to evaluate the prognosis of patients and changes in bacterial resistance patterns. Second, we created models based on past antimicrobial sales using a SARIMA model, and the models did not include unspecific events. The most important unspecific events are the emergence of endemic contagious diseases; nevertheless, no specific contagious diseases, including SARS-CoV-2 became endemic during the study period [27].

## Conclusion

We clarified the effects of the cefazolin shortage on other antimicrobials in terms of sales, costs, and appropriateness of usage. Our study revealed the confusion brought to the antimicrobial market and the worsening of appropriate antimicrobial use due to the cefazolin shortage. Although patients’ prognoses and bacterial resistance patterns should be assessed, our study highlights the need for a framework for risk management against antimicrobial shortages, and for national and international measures for securing the sustainable supply of antimicrobials.

## Supplementary Information

**Additional file 1.**

## Data Availability

The datasets generated and/or analysed during the current study are available in the IQVIA Japan.

## Abbreviations

DID: Defined daily doses/1000 Inhabitants/Day
WHO: World Health Organization
DDD: Defined Daily Dose
SARIMA: Seasonal Autoregressive Integrated Moving Average
USD: United States Dollar
