# Supplementary material for: Effects of the cefazolin shortage on the sales, cost, and appropriate use of other antimicrobials
Source: BMC Health Serv Res. 2021 Oct 19;21:1118. doi: 10.1186/s12913-021-07139-z (PMC8523201; doi:10.1186/s12913-021-07139-z)
Supplement: Supplementary file 1 — Additional file 1. [file 12913_2021_7139_MOESM1_ESM.pdf]

Supplementary materials

Supplementary Figure 1

Dot plots of actual and predicted DID values of antimicrobials

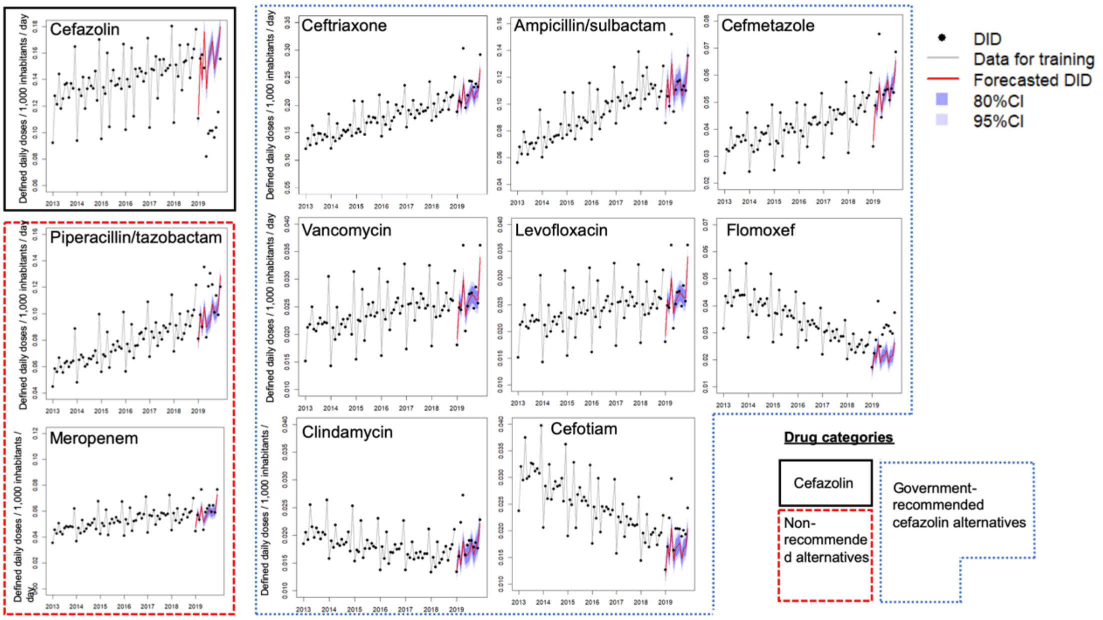

Monthly trends in dot plots of DID values are shown. Red lines represent predictions of DID values in 2019 generated by 2013–2018 data under the assumption of non-occurrence of the cefazolin shortage. Deep purple and light purple zones represent 80% and 95% confidence intervals.

Abbreviation: DID, defined daily doses/1,000 inhabitants/day. CI, confidence intervals.

Supplementary Figure 2

Dot plots of actual and predicted DID values of antimicrobials according to AWaRe classifications

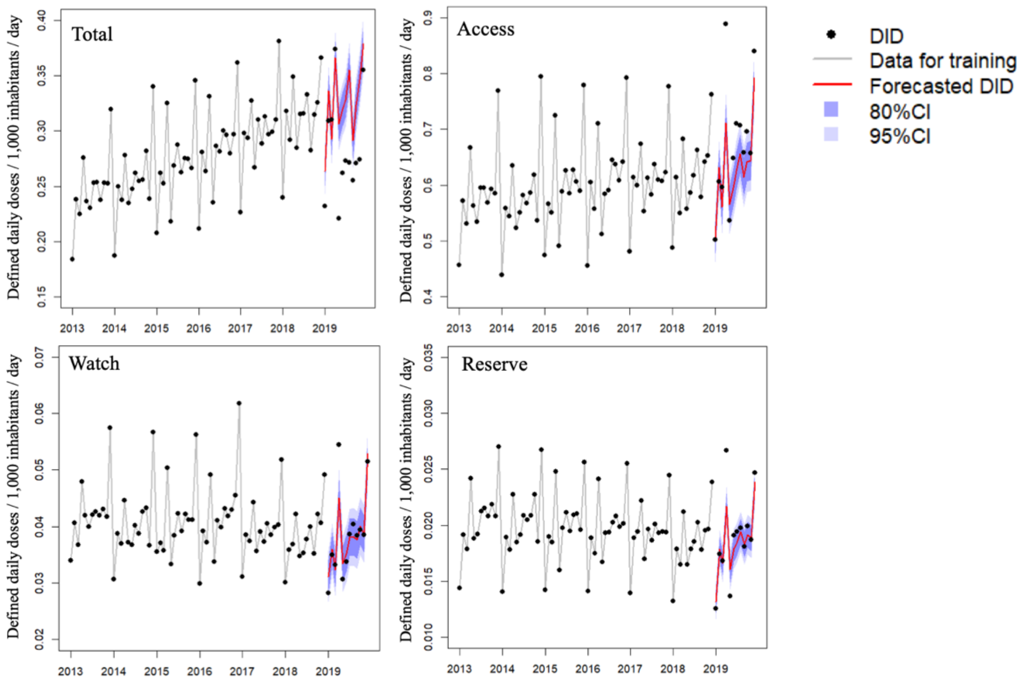

Monthly trends in dot plots of DID values are shown. Red lines represent predictions of DID values in 2019 generated by 2013–2018 data under the assumption of non-occurrence of the cefazolin shortage. Deep purple and light purple zones represent 80% and 95% confidence intervals.

Abbreviation: DID, defined daily doses/1,000 inhabitants/day. CI, confidence intervals.
